# Revision and validation of the screen addiction scale in the Chinese cultural context

**DOI:** 10.1186/s40359-026-04437-1

**Published:** 2026-04-06

**Authors:** Tingting Qiao, Pengzhen Zhou, Xiaodong Wang, Mingmin Wang, Lei Wang

**Affiliations:** 1 Inner Mongolia Medical University, Hohhot, China; 2 Hohhot No.39 Middle School, Hohhot, Inner Mongolia, China; 3 The Second Affifiliated Hospital of Inner Mongolia Medical University, Inner Mongolia, China

**Keywords:** Screen addiction, Chinese cultural context, Reliability and validity, Scale revision and validation

## Abstract

Currently, screen devices have become an integral part of middle school students' daily learning and lives, yet the concomitant issue of screen addiction cannot be overlooked. This study aimed to revise and validate the 21-item Gökçe Screen Addiction Scale within the Chinese cultural context. In Study 1 (*N* = 461), the original scale was adapted into Chinese via forward–backward translation by bilingual experts, followed by pilot testing. This culturally adapted version then underwent preliminary revision. Through item analysis and exploratory factor analysis, one item from the original scale was deleted, and the dimensional structure was adjusted to better align with the screen usage patterns of Chinese adolescents, resulting in a 20-item scale comprising four dimensions. In Study 2 (*N* = 484), the reliability and validity of the preliminarily revised scale were examined. The results of confirmatory factor analysis supported the four-factor structure, demonstrating good construct validity. In Study 3 (*N* = 491), criterion-related validity, convergent validity, discriminant validity, incremental validity, and test–retest reliability were further examined, indicating that the revised scale performed well on all reliability and validity indicators. The final version of the Screen Addiction Scale revised for the Chinese cultural context consists of 20 items across four dimensions: Withdrawal, Mood Regulation, Lying about Use, and Negative Consequences -Compulsive Use. The scale demonstrates good reliability and validity and is suitable for assessing screen addiction levels among Chinese adolescents, thus facilitating targeted interventions for mood regulation and withdrawal in school-based programs.

## Introduction

With the proliferation of digital technology, screen devices have become a significant component of middle school students' daily learning and lives. However, the associated problem of screen addiction is becoming increasingly prominent. Screen addiction refers to an individual's dependence on various screen-based activities and related problematic usage behaviors [1], encompassing engagement in potentially addictive activities involving multiple screen types [2], including televisions, smartphones, tablets, computers, etc. The World Health Organization's report covering 44 countries and regions indicated that in 2022, over one in ten adolescents (11%) exhibited signs of problematic social media use, demonstrating difficulties in controlling their usage and experiencing negative consequences. This issue is also becoming increasingly evident among Chinese adolescents. According to the *Fifth National Survey on Minors' Internet Usage*, a significant proportion of minors exhibit excessive internet use [3]. Specifically, 11.1% of underage netizens reported average daily internet usage exceeding two hours on weekdays, while 13.1% reported averages over five hours per day during holidays. The 2024 Report on Chinese Adolescents' Internet Usage [4] further revealed that the teenage netizen population in China has reached approximately 185 million, with about 13.6% spending over three hours online daily. Additionally, internet usage among minors is trending toward younger age groups. Therefore, the phenomenon of screen addiction among Chinese adolescents warrants serious attention.

Screen addiction poses significant risks to the physical and psychological health, academic performance, and daily functioning of adolescents. Multiple studies have demonstrated that, compared to moderate screen users, individuals with screen addiction are more susceptible to psychological symptoms including insomnia, anxiety, and depression [5–8]. They also show higher vulnerability to physical health issues such as myopia, obesity, cervical spine injuries, musculoskeletal pain, dry eye syndrome, and unhealthy dietary patterns [7, 9–12]. Excessive reliance on screen devices may also contribute to academic procrastination and subsequent decline in academic performance [13, 14]. Furthermore, screen addiction has been significantly associated with aggressive behaviors [15], general bullying [16, 17], as well as cyber aggression and cyber victimization [5]. Longitudinal research further indicates that sustained, prolonged screen use can diminish adolescents' social well-being [18]. Notably, factors such as self-control, psychological resilience, and religious beliefs may serve as important moderators in reducing the risk of screen addiction [19, 20]. In summary, the detrimental effects of screen addiction on adolescent development are substantial, representing an urgent issue that demands immediate attention.

Given the significant and urgent risks associated with adolescent screen addiction, the development of appropriate assessment tools is imperative. To date, international researchers have developed various instruments for evaluating screen addiction, with these tools evolving in parallel with changes in screen devices and usage patterns. Among early contributions, Young [21] developed an 8-item Internet Addiction Diagnostic Questionnaire (IADQ) modeled on pathological gambling criteria, establishing a foundation for subsequent research in this field. With the rising popularity of video games, Lemmens et al. [22] developed the 21-item Game Addiction Scale for Adolescents (GASA), specifically designed to assess computer and video game addiction among adolescents. However, the focus of this scale is relatively narrow, targeting only the single entertainment activity of video games. Meanwhile, the proliferation of smartphones prompted Lin et al. [23] to develop the Smartphone Addiction Inventory (SPAI), which integrates characteristics of internet addiction with smartphone-specific features. This instrument comprises four dimensions: compulsive behavior, functional impairment, withdrawal, and tolerance. Kwon et al. [24] further advanced this area with their 33-item Smartphone Addiction Scale (SAS), encompassing six factors: daily life disruption, withdrawal, positive anticipation, overuse, tolerance, and cyberspace-oriented relationships. While the SAS excels in smartphone specificity, it neglects compulsive use across devices. More recently, Forte et al. [25] addressed emerging binge-watching behaviors through the Binge-Watching Addiction Questionnaire (BWAQ), which distinguishes between pathological viewing patterns and recreational series consumption, thereby filling an important assessment gap in video-based screen addiction. This line of research reveals that addiction symptoms consistently emerge across assessment tools designed for different devices, pointing to an independent syndrome characterized by a generalized screen-based interaction environment as its addictive object and stable symptom expression across devices and contexts. In other words, screen addiction is not merely the sum of various specific addictions, but rather a maladaptive and transferable pattern of dysregulation in an individual’s overall interaction with screen media. Although existing scales do address various forms of screen and internet addiction (e.g., for internet addiction, computer addiction, video game addiction, smartphone addiction, or TV addiction), they remain confined to specific device types and usage contexts, which consequently underscores the integrative advantage of developing a consolidated screen addiction measure. The proliferation of diverse screen devices underscores the need for a comprehensive screen addiction scale, which would offer greater practical utility than developing separate instruments for each screen-based addiction type [1]. Moreover, existing measurement tools—both domestically and internationally—lack a screen addiction scale specifically designed for middle school students. In response to this gap, Gökçe [1] developed a 21-item Screen Addiction Scale for middle school students, comprising four dimensions: Negative Consequences-Compulsive Use, Emotional Regulation, Lying about Use, and Withdrawal-Loss of Interest. This instrument represents a comprehensive tool aimed at assessing various forms of screen and internet addiction, demonstrates good reliability and validity, and effectively measures screen addiction levels among middle school students.

Nevertheless, despite its established strengths, the scale's applicability within the Chinese context may be constrained by differing living environments and cultural backgrounds between Turkish and Chinese adolescents. On one hand, parenting styles in Chinese families frequently emphasize maintaining a balance between screen use and academic responsibilities [26], and parents generally exhibit stricter attitudes regarding screen time management [27]. These contextual factors may contribute to insufficient adaptability of certain scale items when applied to Chinese adolescents. For example, items presuming unrestricted sleep-for-screen trades may underperform amid China's prevalent parental screen-time restrictions [28]. On the other hand, compared to Western populations, Chinese individuals tend to focus more on emotional experiences [29]. This cultural distinction may lead to differential interpretation of item content, potentially resulting in a dimensional structure that does not fully align with the psychological characteristics of Chinese adolescents. Consequently, direct administration of the original scale to this population may introduce cultural bias in measurement outcomes. To address this limitation, the present study builds upon Gökçe’s [1] Screen Addiction Scale by developing and validating a culturally adapted version specifically for Chinese middle school students.

To further examine the predictive validity of the screen addiction scale, this study selected non-suicidal self-injury (NSSI), short-form video addiction, and internet gaming disorder as criterion variables. NSSI refers to the direct and deliberate destruction of one's own body tissue without suicidal intent [30], encompassing behaviors such as cutting, burning, and severe scratching [31]. This condition demonstrates a well-established association with depression [32]. Research has indicated a moderate positive correlation between Internet addiction and NSSI [33], and a causal analysis study has confirmed that increased screen time has a direct causal relationship with NSSI [34]. It is therefore reasonable to postulate that individuals with screen addiction may be at a higher risk of engaging in NSSI compared to those without such addiction. Short-form video addiction represents an emerging form of internet addiction [35], referring to a negative psychological dependence on short-form videos, characterized by an uncontrollable urge to excessively watch them, resulting in physical, psychological, and social functional impairments [36]. The fragmented nature, content richness, and personalized features of short-form videos [36, 37], readily capture and sustain individuals’ attention during screen use, thereby potentially fostering dependence and addictive usage patterns [37]. Internet gaming is one of the primary forms of screen-based engagement among adolescents [5]. Empirical studies demonstrate that internet gaming addiction shows significant positive correlations with extended screen time [38, 39]. Adolescents exhibiting elevated levels of screen addiction may consequently display more severe symptoms of internet gaming addiction. It should be noted that short-form video addiction and internet gaming disorder, as specific manifestations of screen-based behaviors, were included as criterion variables to examine the strength of the association between screen addiction as an independent syndrome and its subordinate addiction types. Meanwhile, non-suicidal self-injury, as a non-screen behavioral outcome, provides validity evidence for the scale beyond screen-specific constructs. Based on this, we hypothesize that screen addiction is significantly positively correlated with NSSI, short-form video addiction, and internet gaming addiction.

In summary, given both the significance of investigating adolescent screen addiction and the cultural adaptation limitations in existing research, this study aims to develop a Screen Addiction Scale appropriate for the Chinese cultural context and to systematically examine its reliability and validity, thereby providing a scientifically validated and effective assessment instrument for measuring screen addiction among Chinese adolescents. The development of this scale is expected to provide a crucial methodological tool for future studies examining risk factors and developmental trajectories of screen addiction, as well as for designing and implementing targeted intervention strategies.

## Study 1: exploratory factor analysis of the screen addiction scale

### Research objective

The purpose of this study was to eliminate items that failed to meet psychometric criteria based on item analysis and to identify the underlying factor structure of the Screen Addiction Scale through exploratory factor analysis, thereby establishing the final version of the scale.

### Participants

The sample size was determined based on the guideline proposed by Hair et al. [40], which recommends that the number of observations should be at least five times the number of variables to be analyzed. Given that the questionnaire comprised 21 items, a minimum of 105 participants was required. The data were collected from middle schools students in two representative cities within XX province of China. With invalidations for > 10% missing or patterned responses (*n* = 6 excluded), the final sample consisted of 461 participants (222 females), aged 12 to 15 years (*M*_age_ = 13.36, *SD*_age_ = 0.69). Among them, 321 were in seventh grade (69.6%), and 140 were in eighth grade (30.4%). The procedures we conducted on human subjects were approved by the ethical standards of the Academic Committee of authors’ University, and conformed to the ethical standards set forth in the 1964 Declaration of Helsinki and its later amendments or similar ethical standards.

### Measurements

The initial version of the Screen Addiction Scale was revised through a rigorous translation and adaptation protocol. The original English instrument developed by Gökçe [1] underwent forward translation by three psychology professionals, followed by back-translation conducted by a bilingual psychology expert to ensure conceptual equivalence. The Chinese version subsequently received psychometric evaluation and refinement, ultimately forming the preliminary scale. Additionally, four experts specializing in adolescent psychological research and six mental health teachers from secondary schools were invited to evaluate the item content. Based on their feedback, the wording of several items was refined to ensure clarity and cultural appropriateness.

This 21-item instrument comprises four dimensions: Withdrawal-Loss of interest (6 items), Mood regulation (4 items), Lying about use (4 items), and Negative consequences-Compulsive use (7 items). A sample item includes: “I would be sad if I had to reduce the time I spend in front of the screen (TV, phone, tablet, computer, etc.)” All items were rated on a 5-point Likert scale from 1 (strongly disagree) to 5 (strongly agree), with higher total scores indicating more severe levels of screen addiction..

### Procedure and data analysis

After obtaining informed consent from the school authorities, homeroom teachers, primary guardians, and the participants themselves, paper-based questionnaires were administered and collected by trained personnel. All subsequent data analyses were performed using SPSS 27.0.

## Results

### Item analysis

Item-total correlation analysis was first conducted to examine the internal consistency of all items. As shown in Table 1, all item-total correlation coefficients exceeded the threshold of 0.4 [41]. Additionally, according to the criterion proposed by DeVellis (2016), inter-item correlations above 0.80 warrant attention for potential multicollinearity issues. In this study, all inter-item correlations were below 0.80 (max = 0.790 for T2-T14), indicating a reduced likelihood of multicollinearity problems [42]. Consequently, all items were retained. In this study, the Cronbach’s α for this scale was 0.960.

**Table 1:** Item-total correlations of the SAS (*r*_*it*_)

Item	*r*_*it*_
T1 I would be sad if I had to reduce the time I spend in front of the screen (TV, smartphone, tablet, computer, etc.)	0.749^***^
T2 I feel nervous when I am not using a screen (TV, smartphone, tablet, computer, etc.)	0.830^***^
T3 I lose sleep to spend time in frontof a screen (TV, smartphone, tablet, computer, etc.)	0.782^***^
T4 The time I spend in front of the screen (TV, smartphone, tablet, computer, etc.) prevents me from spending time with my family	0.643^***^
T5 I have difficulty concentrating on my lessons because I use a screen (TV, smartphone, tablet, computer, etc.)	0.632^***^
T6 I have tried to shorten the time I spend in front of the screen (TV, smartphone, tablet, computer, etc.) but I failed	0.725^***^
T7 I try to hide from my relatives (family or others) how muchtime I spend with the screen (TV, smartphone, tablet, computer, etc.)	0.732^***^
T8 I lie about my responsibilities at home or at school in order to use a screen (TV, smartphone, tablet, computer, etc.)	0.723^***^
T9 There is nothing more fun for me than using screen (TV, smartphone, tablet, computer, etc.)	0.794^***^
T10 Without a screen (TV, smartphone, tablet, computer, etc.), my life would be empty	0.850^***^
T11 I spend time secretly in front of the screen (TV, smartphone, tablet, computer, etc.)	0.775^***^
T12 I postpone the work I need to do in order to use a screen (TV, smartphone, tablet, computer, etc.)	0.783^***^
T13 I feel pleasant when I spend time with a screen (TV, smartphone, tablet, computer, etc.)	0.689^***^
T14 I get stressed when I am not using a screen(TV, smartphone, tablet, computer, etc.)	0.849^***^
T15 Nothing makes me happy except using a screen (TV, smartphone, tablet, computer, etc.)	0.753^***^
T16 Immediately after I stop using a screen (TV, smartphone, tablet, computer, etc.), I feel the need to use it again	0.843^***^
T17 I feel better when I use a screen (TV, smartphone, tablet, computer, etc.)	0.753^***^
T18 When I'm having a bad day, it seems like the only thing that can lift my mood is using a screen (TV, smartphone, tablet, computer, etc.)	0.734^***^
T19 It's becoming increasingly difficult for me to stop using screens (TV, smartphone, tablet, computer, etc.)	0.789^***^
T20 It is possible to get rid of stress by using a screen (TV, smartphone, tablet, computer, etc.)	0.631^***^
T21 I can lie to my family to spendtime in front of a screen (TV, smartphone, tablet, computer, etc.)	0.684^***^

^***^p <.001

Subsequently, independent samples t-tests were conducted for each item comparing the high screen addiction group (top 27%) and the low screen addiction group (bottom 27%). The results indicated statistically significant differences across all items, with t-values ranging from 12.752 to 23.313 (all *p*s < 0.001), demonstrating the scale's adequate discriminant validity.

#### Exploratory factor analysis and reliability analysis

Given that this study aimed to uncover the latent structure underlying the variables and adhered to contemporary psychometric scale development standards, Principal Axis Factoring (PAF) was employed in the exploratory factor analysis to extract four factors [43, 44]. Furthermore, given the correlations among the items, an optimal oblique rotation method (promax) was selected [43, 44]. The Kaiser–Meyer–Olkin (KMO) measure reached 0.955, and Bartlett's test of sphericity yielded significant results (χ^2^ = 7763.196, df = 210, *p* < 0.001), confirming the data's suitability for factor analysis. Factor loadings are detailed in Table 2. The item exclusion criteria included: (1) items with communalities below 0.40; (2) factor loadings less than 0.30; and (3) cross-loading on multiple factors; and (4) fewer than three items per factor [40, 43, 44]. Following these criteria, item T3 was eliminated, resulting in a final 20-item instrument.

**Table 2:** Principal component analysis (21 items)

Item	1	2	3	4	Communalities
T2 I feel nervous when I am not using a screen (TV, smartphone, tablet, computer, etc.)	0.493				0.772
T9 There is nothing more fun for me than using screen (TV, smartphone, tablet, computer, etc.)	0.670				0.668
T10 Without a screen (TV, smartphone, tablet, computer, etc.), my life would be empty	0.639				0.762
T14 I get stressed when I am not using a screen(TV, smartphone, tablet, computer, etc.)	0.691				0.774
T15 Nothing makes me happy except using a screen (TV, smartphone, tablet, computer, etc.)	1.023				0.747
T16 Immediately after I stop using a screen (TV, smartphone, tablet, computer, etc.), I feel the need to use it again	0.472				0.728
T19 It's becoming increasingly difficult for me to stop using screens (TV, smartphone, tablet, computer, etc.)	0.341				0.604
T1 I would be sad if I had to reduce the time I spend in front of the screen (TV, smartphone, tablet, computer, etc.)		0.467			0.641
T13 I feel pleasant when I spend time with a screen (TV, smartphone, tablet, computer, etc.)		0.935			0.671
T17 I feel better when I use a screen (TV, smartphone, tablet, computer, etc.)		0.797			0.700
T18 When I'm having a bad day, it seems like the only thing that can lift my mood is using a screen (TV, smartphone, tablet, computer, etc.)		0.554			0.620
T20 It is possible to get rid of stress by using a screen (TV, smartphone, tablet, computer, etc.)		0.818			0.543
T7 I try to hide from my relatives (family or others) how much time I spend with the screen (TV, smartphone, tablet, computer, etc.)			0.630		0.642
T8 I lie about my responsibilities at home or at school in order to use a screen (TV, smartphone, tablet, computer, etc.)			0.578		0.638
T11 I spend time secretly in front of the screen (TV, smartphone, tablet, computer, etc.)			0.742		0.733
T12 I postpone the work I need to do in order to use a screen (TV, smartphone, tablet, computer, etc.)			0.515		0.649
T21 I can lie to my family to spend time in front of a screen (TV, smartphone, tablet, computer, etc.)			0.683		0.587
T4 The time I spend in front of the screen (TV, smartphone, tablet, computer, etc.) prevents me from spending time with my family				0.805	0.578
T5 I have difficulty concentrating on my lessons because I use a screen (TV, smartphone, tablet, computer, etc.)				0.663	0.542
T6 I have tried to shorten the time I spend in front of the screen (TV, smartphone, tablet, computer, etc.) but I failed				0.557	0.575
T3 I lose sleep to spend time in front of a screen (TV, smartphone, tablet, computer, etc.)				0.278	0.599

Principal component analysis with promax rotation was reapplied to the remaining 20 items, yielding a four-factor solution (see Table 3). The Kaiser–Meyer–Olkin (KMO) measure was 0.954, and Bartlett's test of sphericity reached statistical significance (*χ*^*2*^ = 7307.222, *df* = 190, *p* < 0.001). These four factors collectively accounted for 65.884% of the total variance. Factor 1, labeled 'Withdrawal-Loss of Interest', comprised seven items (2, 9, 10, 14, 15, 16, 19) describing physical or psychological discomfort following sudden reduction or discontinuation of screen use, with factor loadings ranging from 0.338 to 1.018. Factor 2, designated 'Mood Regulation', contained five items (1, 13, 17, 18, 20) reflecting the tendency to utilize screens for alleviating negative emotional states, with factor loadings ranging from 0.474 to 0.922. Factor 3, termed 'Lying about Use', included five items (7, 8, 11, 12, 21) addressing deceptive behaviors regarding screen usage patterns to significant others (e.g., family, friends), with factor loadings ranging from 0.509 to 0.744. Factor 4, identified as 'Negative Consequences-Compulsive Use', consisted of three items (4, 5, 6) capturing persistent screen use despite adverse repercussions, with factor loadings ranging from 0.561 to 0.796. In comparison with Gökçe's original scale, several items demonstrated differential factor loadings in the present study: item T1 was reassigned from the Withdrawal dimension to Mood Regulation; item T12 shifted from the Negative consequences-Compulsive use dimension to Lying about use; and items T16 and T19 moved from the Negative consequences–Compulsive use dimension to Withdrawal-Loss of Interest. For the final 20-item Screen Addiction Scale, the Cronbach's α coefficients were 0.958 for the total scale, and 0.940, 0.876, 0.893, and 0.792 for the Withdrawal-Loss of Interest, Mood Regulation, Lying about Use, and Negative Consequences-Compulsive Use dimensions, respectively.

**Table 3:** Principal component analysis (20 items)

Item	1	2	3	4	Communalities
T2 I feel nervous when I am not using a screen (TV, smartphone, tablet, computer, etc.)	0.510				0.760
T9 There is nothing more fun for me than using screen (TV, smartphone, tablet, computer, etc.)	0.668				0.662
T10 Without a screen (TV, smartphone, tablet, computer, etc.), my life would be empty	0.637				0.764
T14 I get stressed when I am not using a screen(TV, smartphone, tablet, computer, etc.)	0.693				0.777
T15 Nothing makes me happy except using a screen (TV, smartphone, tablet, computer, etc.)	1.108				0.752
T16 Immediately after I stop using a screen (TV, smartphone, tablet, computer, etc.), I feel the need to use it again	0.474				0.726
T19 It's becoming increasingly difficult for me to stop using screens (TV, smartphone, tablet, computer, etc.)	0.338				0.608
T1 I would be sad if I had to reduce the time I spend in front of the screen (TV, smartphone, tablet, computer, etc.)		0.474			0.630
T13 I feel pleasant when I spend time with a screen (TV, smartphone, tablet, computer, etc.)		0.922			0.668
T17 I feel better when I use a screen (TV, smartphone, tablet, computer, etc.)		0.788			0.702
T18 When I'm having a bad day, it seems like the only thing that can lift my mood is using a screen (TV, smartphone, tablet, computer, etc.)		0.556			0.619
T20 It is possible to get rid of stress by using a screen (TV, smartphone, tablet, computer, etc.)		0.815			0.547
T7 I try to hide from my relatives (family or others) how much time I spend with the screen (TV, smartphone, tablet, computer, etc.)			0.620		0.640
T8 I lie about my responsibilities at home or at school in order to use a screen (TV, smartphone, tablet, computer, etc.)			0.569		0.632
T11 I spend time secretly in front of the screen (TV, smartphone, tablet, computer, etc.)			0.744		0.734
T12 I postpone the work I need to do in order to use a screen (TV, smartphone, tablet, computer, etc.)			0.509		0.643
T21 I can lie to my family to spend time in front of a screen (TV, smartphone, tablet, computer, etc.)			0.680		0.591
T4 The time I spend in front of the screen (TV, smartphone, tablet, computer, etc.) prevents me from spending time with my family				0.796	0.584
T5 I have difficulty concentrating on my lessons because I use a screen (TV, smartphone, tablet, computer, etc.)				0.670	0.554
T6 I have tried to shorten the time I spend in front of the screen (TV, smartphone, tablet, computer, etc.) but I failed				0.561	0.583

### Summary

A 20-item Screen Addiction Scale was finalized through exploratory factor analysis following the removal of one item (T3). The scale comprises four dimensions based on item content: Withdrawal-Loss of Interest (7 items), Mood regulation (5 items), Lying about use (5 items), and Negative consequences-Compulsive use (3 items). The scale demonstrated high internal consistency with a Cronbach's α coefficient of 0.958.

## Study 2: confirmatory factor analysis of screen addiction scale

### Participants

Consistent with the participant selection criteria used in Study 1, the sample size for this study was determined based on the recommendation of Hair et al. [40], which required a minimum of 100 participants. The data were collected from middle schools students in two representative cities within XX province of China. The sample was independent of Study 1, and no questionnaires needed to be excluded due to > 10% missing or patterned responses. The final sample comprised 484 participants (240 females), aged 12 to 15 years (*M*_age_ = 13.35, *SD*_age_ = 0.67). This sample size yields > 95% power for detecting an RMSEA < 0.08 [45]. Among them, 343 were in seventh grade (70.9%), and 141 were in eighth grade (29.1%). The procedures we conducted on human subjects were approved by the ethical standards of the Academic Committee of authors’ University, and conformed to the ethical standards set forth in the 1964 Declaration of Helsinki and its later amendments or similar ethical standards.

### Measurements

#### The revised screen addiction scale

The revised Screen Addiction Scale was used with 20 items and contained 4 dimensions: withdrawal-loss of interest (7 items), mood regulation (5 items), lying about use (5 items), and negative consequences-compulsive use (3 item). Example item: “I would be sad if I had to reduce the time I spend in front of the screen (TV, phone, tablet, computer, etc.).” The scale adopted 5-point score, where 1 = strongly disagree, 5 = strongly agree. Higher scores indicated greater screen addiction. The Cronbach's α of the scale in this study was 0.955.

### Procedures and statistical analysis

After obtaining the informed consent of the school, the class teacher, the main guardian and the individual, the electronic questionnaires will be distributed and collected by professionals on a class basis. All analyses were performed using SPSS 27.0 and M-plus 8.0.

### The results of confirmatory factor analysis

According to the results of the exploratory factor analysis, the screen addiction scale was designed to comprise four dimensions. A structural equation modeling (SEM) approach was then employed for confirmatory factor analysis (CFA). Model fit was assessed using multiple indices: the χ^2^/df ratio, Tucker-Lewis index (TLI), comparative fit index (CFI), standardized root mean square residual (SRMR), and root mean square error of approximation (RMSEA). The results indicated an acceptable model fit (χ^2^/df = 3.258, RMSEA = 0.068, CFI = 0.951, TLI = 0.940, SRMR = 0.041), which was significantly better than that of alternative models. These findings further support the four-dimensional structure of the scale, demonstrating that the screen addiction scale has good construct validity (see Table 4).

**Table 4:** The results of confirmatory factor analysis

	χ^2^*/df*	RMSEA	CFI	TLI	SRMR
Four-factor model	3.258^***^	0.068	0.951	0.940	0.041
Three-factor model	4.792^***^	0.089	0.912	0.899	0.052
Two-factor model	6.294^***^	0.105	0.877	0.859	0.060
One-factor model	7.514^***^	0.116	0.847	0.827	0.062

^***^p <.001; Four-factor model: A、B、C、D; Three-factor model: A、B、C + D; Two-factor model: A、B + C + D; One-factor model: A + B + C + D

### Summary

The results of confirmatory factor analysis showed that the screen addiction scale included four dimensions of withdrawal-loss of interest, mood regulation, lying about use, and negative consequences-compulsive use, and the Cronbach's α of the scale was 0.955. This indicates that the scale has good construct validity and reliability and can be used in subsequent research.

## Study 3: reliability and validity testing of screen addiction scale

### Participants

Criterion-related validity sample: Based on Hair’s (2019) suggestion of a minimum sample size of 200 for correlational research, and after invalidating questionnaires with > 10% missing or patterned responses (*n* = 9 excluded), the final sample comprised of 491 participants (233 females) from middle schools in two representative cities within XX Province of China. The sample was recruited independently but remained demographically comparable to those in Studies 1 and 2 (χ^2^ test for grade equivalence, *p* = 0.088). Participants were aged 12 to 15 years (*M*_age_ = 13.31, *SD*_age_ = 0.66). Among them, 366 were in seventh grade (74.5%), and 125 were in eighth grade (25.5%).

Convergent, discriminant, and incremental validity sample: A total of 315 participants (138 females) from middle schools in two representative cities within XX Province of China were selected. No questionnaires had > 10% missing data or patterned responses. Participants were aged 13 to 15 years, *M*_age_ = 13.81, *SD*_age_ = 0.62.

Test–retest reliability sample: After a two-week interval, participants from the previous sample were invited for test–retest reliability assessment. No questionnaires had > 10% missing data or patterned responses. The final sample comprised 138 participants (56 females) from middle schools in two representative cities within XX Province of China, aged 13 to 15 years, *M*_age_ = 13.88, *SD*_age_ = 0.55.

The procedures we conducted on human subjects were approved by the ethical standards of the Academic Committee of authors’ University, and conformed to the ethical standards set forth in the 1964 Declaration of Helsinki and its later amendments or similar ethical standards.

### Measurements

#### Screen addiction scale

The revised Screen Addiction Scale was used with 20 items and contained 4 dimensions: withdrawal-loss of interest (7 items), mood regulation (5 items), lying about use (5 items), and negative consequences-compulsive use (3 item). Example item: "I would be sad to if I had to reduce the time I spend in front of a screen (TV, phone, tablet, computer, etc.)." The scale adopted 5-point score, where 1 = strongly disagree, 5 = strongly agree. Higher scores indicated greater screen addiction. In the three samples of this study, Cronbach's α for this scale was 0.949, 0.956, and 0.967, respectively.

#### Deliberate self-harm inventory

The frequency of non-suicidal self-injury (NSSI) behavior was measured by the deliberate self-harm inventory developed by Gratz [46] and revised by Huang et al. [47]. The scale has 11 items, for example, "Severely scratched yourself, to the extent that scarring or bleeding occurred?" The scale uses a 6-point score, where 1 = 0 times, 2 = 1 times, 3 = 2 times, 4 = 3 times, 5 = 4 times, 6 = 5 times and more, and the higher the score, the higher the frequency of NSSI of the individual. The Cronbach's α of the scale in this study was 0.890.

#### Short video APP addiction scale

Based on the Bergen Facebook Addiction Scale developed by Andreassen et al. [48] and related theoretical framework, it is revised by combining the motivation characteristics of short video addiction to measure the degree of short video addiction of individuals. The revised scale consists of 10 items, for example, "When I cannot use the short video APP, I feel anxious or annoyed." The scale adopted a 5-point score, where 1 = very rarely, 5 = often, with higher scores indicating more severe short video addiction. The Cronbach's α of the scale in this study was 0.911.

#### Internet game addiction scale

The Internet Game Addiction scale developed by Gentile [49] and revised by Yu et al. [50] was used to measure the degree of individuals’ internet game addiction. The scale consists of 11 items, such as "Do you feel that you need to play more and more internet games to be satisfied?" Participants rate each item from 1 (never) to 3 (often), with higher scores indicating more serious levels of online game addiction. In this study, the Cronbach's α of the scale was 0.867.

#### Brief Self-Control Scale (BSCS)

Self-control was measured using the Brief Self-Control Scale developed by Tangney et al. [51] and revised by Morean (2014), for convergent validity testing. The 7-item scale includes the item: "I am good at resisting temptation." Items 2, 4, 6, and 7 were reverse-scored. The scale used a 5-point Likert scale, where 1= not at all, 2 = somewhat not, 3 = uncertain, 4 = somewhat, and 5 = completely. After reverse scoring, higher scores indicated greater self-control. In this study, Cronbach's α was 0.680.

#### Brief Barratt Impulsiveness Scale (BBIS)

Impulsivity was measured using the Brief Barratt Impulsiveness Scale developed by Morean et al. [52] and translated and validated by Luo et al. [53], for convergent validity testing. The 8-item scale includes the item: "I do things without thinking." Items 1, 4, 5, and 6 were reverse-scored. The scale used a 4-point rating scale, where 1 = never, 2 = occasionally, 3 = sometimes, and 4 = often. After reverse scoring, higher scores indicated greater impulsivity. In this study, Cronbach's α was 0.803.

#### Mental health inventroy of middle-school students (MMHI-60)

Depression, anxiety, and academic stress were measured using the corresponding subscales from the Mental Health Inventory for Middle School Students developed by Wang et al. [54], for discriminant validity testing. The depression subscale comprises 5 items (e.g., "I feel depressed"), the anxiety subscale comprises 6 items (e.g., "I feel nervous or easily tense"), and the academic stress subscale comprises 6 items (e.g., "I feel that my academic burden is heavy"). The scale used a 5-point rating scale, where 1 = none, 2 = mild, 3 = moderate, 4 = relatively severe, and 5 = severe. Higher scores indicated greater severity of mental health problems in the corresponding dimension. In this study, Cronbach's α was 0.928 for depression, 0.949 for anxiety, and 0.920 for academic stress.

#### Young's Internet Addiction Test (IAT)

Internet addiction was measured using Young's Internet Addiction Test developed by Young [55], for incremental validity testing. This 20-item scale includes the item: "Do you find that you stay online longer than you intended?" The scale used a 5-point rating scale, where 1 = rarely, 2 = occasionally, 3 = sometimes, 4 = often, and 5 = always. Higher scores indicated more severe internet addiction. In this study, Cronbach's α was 0.964.

### Procedures and statistical analysis

After obtaining the informed consent of the school, the class teacher, the main guardian and the individual, the electronic questionnaires will be distributed and collected by professionals on a class basis. All analyses were performed using SPSS 27.0 and Mplus 8.0.

### Results

#### Criterion-related validity results

The correlations among screen addiction, NSSI, short video addiction, and internet game addiction were shown in Table 5. The results showed that screen addiction was positively correlated with NSSI (*r* = 0.352, 95% CI [0.210, 0.441], *p* < 0.001), short video addiction (*r* = 0.622, 95% CI [0.519, 0.703], *p* < 0.001), and internet game addiction (*r* = 0.570, 95% CI [0.469, 0.664], *p* < 0.001). These associations remained robust after controlling for age and gender, as indicated by the partial correlation coefficients (with NSSI: *pr* = 0.317, *p* < 0.001; with short video addiction: *pr* = 0.615, *p* < 0.001; with internet game addiction: *pr* = 0.597, *p* < 0.001). This indicated that the criterion-related validity of the screen addiction scale is good.

**Table 5:** The correlations among screen addiction, NSSI, short video addiction, and internet game addiction

	*M*	*SD*	1	2	3	4
1 NSSI	0.245	0.617	-			
2 Short Video Addiction	2.454	0.976	0.290^***^	-		
3 Internet Game Addiction	1.369	0.369	0.264^***^	0.495^***^	-	
4 Screen Addiction	2.139	0.795	0.352^***^	0.622^***^	0.570^***^	-

*N* = 491. ^***^p < 0.001

#### Convergent validity, discriminant validity, and incremental validity results

The correlations among screen addiction, self-control, impulsivity, depression, anxiety, and academic stress are shown in Table 6. The results showed that screen addiction had a moderately strong significant negative association with self-control (*r* = −0.534, 95% CI [−0.609, −0.450], *p* < 0.001) and a moderately strong significant positive association with impulsivity (*r* = 0.536, 95% CI [0.452, 0.610], *p* < 0.001), indicating good convergent validity of the Screen Addiction Scale. Additionally, screen addiction showed moderately weak significant positive associations with depression (*r* = 0.375, 95% CI [0.276, 0.467], *p* < 0.001), anxiety (*r* = 0.329, 95% CI [0.227, 0.424], *p* < 0.001), and academic stress (*r* = 0.403, 95% CI [0.306, 0.492], *p* < 0.001), indicating good discriminant validity of the Screen Addiction Scale.

**Table 6:** The correlations among screen addiction, self-control, impulsivity, depression, anxiety, and academic stress

	*M*	*SD*	1	2	3	4	5	6
1 self-control	3.472	0.685	-					
2 impulsivity	2.141	0.577	−0.702^***^	-				
3 depression	1.839	0.989	−0.444^***^	0.413^***^	-			
4 anxiety	1.830	0.960	−0.351^***^	0.355^***^	0.863^***^	-		
5 academic stress	2.149	1.013	−0.429^***^	0.421^***^	0.722^***^	0.748^***^	-	
6 Screen Addiction	2.244	0.814	−0.534^***^	0.536^***^	0.375^***^	0.329^***^	0.403^***^	-

*N* = 315. ^***^p < 0.001

This study examined the incremental validity of the Screen Addiction Scale relative to Young's Internet Addiction Test (IAT) in predicting impulsivity. The results showed that after controlling for Young's Internet Addiction Test, the inclusion of the Screen Addiction Scale in the equation accounted for an additional 3.00% of the variance in impulsivity (β = −0.223, *p* = 0.001; Δ*R*^2^ = 0.030), indicating that the Screen Addiction Scale has good incremental validity relative to Young's Internet Addiction Test (Hunsley & Meyer, 2003).

#### Test–retest reliability results

The test–retest reliability results showed that the test–retest reliability coefficient for the Screen Addiction Scale was 0.700, exceeding the criterion of 0.600 [56], indicating that the Screen Addiction Scale has acceptable test–retest reliability.

### Summary

The criterion-related validity analysis demonstrated that screen addiction was significantly positively correlated with short-form video addiction, internet gaming addiction, and non-suicidal self-injury (NSSI), indicating that individuals with higher levels of screen addiction may have greater tendencies toward short-form video and internet gaming addiction, and may exhibit more non-suicidal self-injury behaviors. Furthermore, screen addiction showed relatively high significant correlations with self-control and impulsivity, and relatively lower but significant correlations with depression, anxiety, and academic stress. Additionally, the Screen Addiction Scale demonstrated incremental validity relative to Young's Internet Addiction Test. The test–retest reliability coefficients for the total score and subscales of the Screen Addiction Scale were also satisfactory. These findings suggest that the Screen Addiction Scale possesses good criterion-related validity, convergent validity, discriminant validity, and test–retest reliability, supporting its utility in future research.

## Discussion

This study aimed to revise the Screen Addiction Scale for the Chinese cultural context, resulting in a 20-item version modified from Gökçe’s [1] original instrument. Both exploratory and confirmatory factor analyses demonstrated satisfactory psychometric properties, supporting a four-dimensional structure comprising: Withdrawal-Loss of Interest, Mood Regulation, Lying About Use, and Negative Consequences-Compulsive Use. All dimensions exhibited adequate internal consistency reliability.

According to the results of an exploratory factor analysis, the Screen Addiction Scale in the Chinese context comprises four dimensions: Withdrawal-Loss of Interest (7 items), Mood Regulation (5 items), Lying about Use (5 items), and negative consequences-compulsive use (3 items). These four dimensions accounted for 72.821% of the total variance, All 20 items demonstrated acceptable factor loadings ranging from 0.651 to 0.939.

Item 3, "I would sacrifice sleep for time spent in front of a screen (TV, smartphone, tablet, computer, etc.)," was eliminated due to its factor loading falling below the 0.40 threshold. This phenomenon may be attributed to stricter parental controls over electronic device usage among Chinese adolescents [27]. As parents typically impose limitations on screen time, participants rarely sacrifice sleep for screen-based activities, thereby reducing the item's discriminative capacity within Chinses cultural context. Consequently, this item was excluded to enhance the scale's ecological validity for Chinese adolescents.

Based on the results of exploratory factor analysis, this study reallocated items T1, T12, T16, and T19 to different dimensions. Specifically, item T1—" I would feel sad if I had to spend less time in front of screens (TV, smartphones, tablets, computers, etc.)"—was relocated from the original Withdrawal-Loss of Interest dimension to Mood Regulation. This shift may reflect cultural differences between Chinese and Western populations. Previous research indicates that Chinese individuals tend to focus on potential negative outcomes of positive events [57] and frequently employ mood regulation strategies [58]. Consequently, participants' attention was likely drawn to the emotional cue "sad" in the item, leading them to interpret it through the lens of mood regulation rather than withdrawal symptoms. Item T12, " I postpone the work I need to do in order to use a screen (TV, smartphone, tablet, computer, etc.)." was reassigned from the original Negative Consequences-Compulsive Use dimension to the Lying about Use dimension. This dimensional shift may reflect the dual regulatory environment surrounding Chinese adolescents' screen use, characterized by restrictions both at home and in school contexts [27, 59]. Previous research indicates that procrastination often serves as an indirect strategy to negotiate screen time amidst such external controls [60, 61], a behavioral pattern frequently accompanied by concealed usage behaviors [62]. Consequently, within the Chinese adolescent sample, this item exhibited stronger psychometric alignment with the Lying About Use dimension. Items T16 and T19 were reassigned from the original Negative Consequences-Compulsive Use dimension to the Withdrawal-Loss of Interest dimension. This dimensional transition may be associated with Chinese adolescents' heightened sensitivity to emotional experiences within their cultural context [29]. According to the Screen Addiction Scale, the Negative Consequences-Compulsive Use dimension primarily addresses the external functional impairments resulting from screen addiction (e.g., familial conflicts or academic disruption), whereas Withdrawal-Loss of Interest captures the physiological and psychological discomfort arising from abstinence or reduced usage [1]. In contrast to other items in the Negative Consequences-Compulsive Use dimension, these two items specifically capture immediate psychological and behavioral responses to screen cessation rather than chronic functional impairments. This formulation led Chinese adolescents to predominantly focus on the negative emotional experiences associated with discontinuing screen use—a conceptual alignment with the core characteristics of withdrawal. The differential factor loadings observed between Chinese and Western samples reflect fundamental distinctions in cultural frameworks.

Additionally, this study selected non-suicidal self-injury (NSSI), short-form video addiction, and internet gaming disorder as criterion variables, employing three psychometrically validated instruments for assessment. The results revealed significant positive correlations between the Screen Addiction Scale and all three criterion measures, demonstrating adequate criterion-related validity. Notably, short-form video addiction and internet gaming disorder, as specific manifestations of screen-based behaviors, showed moderate positive correlations with screen addiction. This pattern aligns with the relationship between a higher-order syndrome and its subordinate subtypes. Meanwhile, the significant positive correlation between screen addiction and NSSI, a non-screen behavioral outcome, indicates that the scale is effectively associated with clinical consequences beyond the screen behavior domain, thus providing criterion-related validity evidence that extends beyond screen-specific constructs. Specifically, NSSI is defined as the direct and deliberate destruction of one's body tissue without suicidal intent [30]. Previous research has established a significant positive association between increased screen time and non-suicidal self-injury (NSSI) behaviors [34]. Consistent with prior literature, the current findings show that higher levels of screen addiction correspond to increased severity of NSSI [33]. The study further revealed that elevated levels of screen addiction were associated with increased severity of short-form video addiction. As an emerging modality of online information dissemination, short-form videos have gained popularity due to their fragmented nature, embeddedness in daily life, and content richness [36], establishing this behavioral pattern as a distinct variant of internet addiction [35]. Consequently, individuals with screen addiction, through their frequent device engagement, demonstrate heightened susceptibility to the captivating features of short-form videos, potentially developing compulsive usage patterns characteristic of this specific behavioral addiction. Furthermore, our study also found that there was a positive correlation between screen addiction and online game addiction, which was consistent with previous findings (Columb, 2024); [39]. Recent research indicates progressively earlier initial exposure to electronic screens among adolescents, accompanied by steadily increasing screen time utilization [63]. Given that internet gaming represents one of the primary forms of screen-based engagement for this demographic [5], adolescents exhibiting elevated screen addiction levels consequently demonstrate more severe manifestations of internet gaming disorder. These findings collectively demonstrate that screen addiction shows significant positive associations with non-suicidal self-injury, short-form video addiction, and internet game addiction. These cross-sectional associations provide an empirical foundation for future longitudinal research to explore potential causal pathways among these variables.

In summary, the Screen Addiction Scale comprises 20 items across four dimensions: Withdrawal-Loss of Interest, Mood Regulation, Lying About Use, and Negative Consequences-Compulsive Use. The revision process involved the removal of one item from the original Negative Consequences-Compulsive Use dimension and the reassignment of four items to different dimensions. These modifications have enhanced the scale's alignment with Chinese cultural context, thereby improving its accuracy and reliability in assessing screen addiction among Chinese adolescents. The adapted instrument more effectively reflects participants' authentic experiences and provides researchers with a more applicable assessment tool.

However, this study has several limitations. First, all variables were assessed exclusively through self-report measures, which may introduce common method bias. It has been noted that self-reported cognitive measures may vary by age and gender [64], suggesting the need for multi-informant approaches (e.g., reports from teachers or parents) in evaluating adolescent screen addiction, alongside objective measurement methods (e.g., cortisol levels or other physiological markers). Future research should adopt multi-wave longitudinal designs to more rigorously examine the developmental trajectories of screen addiction and their dynamic relationships with related variables. Second, the sample was recruited from a single province in China, which may introduce sampling bias. However, as the participants were drawn from different middle schools in two representative cities, the sample demonstrates reasonable representativeness. Subsequent studies should employ multi-regional sampling strategies and examine how demographic factors (e.g., urban–rural residency and socioeconomic status) may moderate the observed relationships. Third, regulatory norms such as curfews may have created a floor effect on compulsivity scores, while social norms likely led to range restriction in the Lying dimension. Future studies should consider using normed scoring to mitigate these scale-specific constraints.

## Conclusion

The revised Screen Addiction Scale contains four dimensions: Withdrawal-Loss of Interest, Mood Regulation, Lying About Use, and Negative Consequences-Compulsive Use, comprising 20 items in total. This instrument demonstrates good reliability and validity as a measurement tool for assessing screen addiction. As the original scale did not provide clinical cutoffs, this preliminary validation study with Chinese populations has not yet established such thresholds. Future research may employ ROC curve analysis in conjunction with clinical interviews or DSM-5 criteria to derive clinically meaningful cutoffs for screening and triage purposes. It is currently best suited for use with individuals aged 12–15 years, with potential for extension to the broader adolescent student population. If applied to younger groups such as elementary school students, measurement invariance testing is recommended.

## Data Availability

The datasets used and/or analysed during the current study are available from the corresponding author on reasonable request.
